# Evaluating Chronic Sex-Specific Changes in Glutamatergic Signaling Markers Following Traumatic Brain Injury

**DOI:** 10.3390/ijms27062670

**Published:** 2026-03-14

**Authors:** Caiti-Erin Talty, Madison S. Wypyski, Susan F. Murphy, Pamela J. VandeVord

**Affiliations:** 1 Graduate Program in Translational Biology, Medicine & Health, Virginia Tech, 325 Stanger St., Blacksburg, VA 24061, USA; caitierin@vt.edu (C.-E.T.); madisonw23@vt.edu (M.S.W.); 2 Department of Biomedical Engineering, Virginia Tech, 325 Stanger St., Blacksburg, VA 24061, USA; murphysf@vt.edu; 3 Veterans Affairs Medical Center, 1970 Roanoke Blvd, Salem, VA 24153, USA

**Keywords:** traumatic brain injury, glutamate, concussion, synapse, NMDA receptor, sex-specific outcomes

## Abstract

Traumatic brain injury (TBI) can lead to persistent adverse outcomes, including cognitive and emotional dysfunction, with recent estimates indicating that up to 50% of individuals with mild TBI experience long-term symptoms. Growing evidence suggests that biological sex influences TBI outcomes and recovery trajectories; however, the molecular underpinnings driving these sex-specific differences remain poorly understood. In this study, a preclinical TBI model was used to directly compare chronic glutamatergic alterations in adult male and female Sprague Dawley rats. To define frontocortical molecular signatures associated with sex-specific glutamatergic dysfunction, proteomic analyses were conducted. Proteomic data revealed dysregulation of key pathways, cellular processes, and molecular regulators involved in excitatory signaling and synaptic function in both sexes. Biomarker profiling identified a single common biomarker between males and females, along with multiple biomarkers unique to each sex. Furthermore, two key brain regions highly susceptible to TBI, the prefrontal cortex and hippocampal subregions, were examined for chronic alterations in key glutamatergic signaling proteins, including N-methyl-D-aspartate (NMDA) receptors and the excitatory synaptic marker postsynaptic density protein 95 (PSD95). Immunofluorescence analyses revealed both sex- and region-specific alterations in the expression of NMDA receptor subunits, as well as in PSD95. Notably, many of these changes were concentrated within the hippocampal subregions, suggesting long-term dysregulation of hippocampal glutamatergic circuitry following injury. Together, these findings indicate the emergence of chronic sex-specific pathophysiology in glutamate signaling after TBI and highlight the importance of incorporating sex as a biological variable in the development of precision medicine-based therapeutic strategies for TBI.

## 1. Introduction

Mild traumatic brain injury (mTBI) affects an estimated 80% of the 69 million people who suffer brain injuries worldwide each year [1]. Although this type of injury is termed “mild,” recent longitudinal studies have reported that over 50% of patients may suffer from persistent adverse symptoms, including somatic complaints, mental health challenges, and cognitive problems [2,3,4]. Clinically, men and women tend to exhibit different recovery patterns, with females reporting more severe post-concussion symptoms and longer recovery times than males [5,6,7]. However, preclinical studies examining sex differences in TBI outcomes are limited and have produced inconsistent findings. Some studies report greater resilience in females, whereas others identify increased behavioral deficits in females or observe no significant sex-based differences in recovery [8,9,10]. Potential contributors to this heterogeneity in sex difference findings include methodological variations and a lack of standardization across preclinical models, which complicate interpretation and limit translational relevance [11].

Sex differences in behavioral outcomes following TBI may reflect underlying sex-specific pathophysiological mechanisms. Immediately following injury, excessive glutamate is released into the extracellular space, initiating excitotoxic cascades that can lead to neuronal dysfunction and cell death [12]. Emerging evidence suggests that the neurons that survive this acute excitotoxic insult may still undergo persistent functional and molecular alterations [13,14]. Despite these findings, long-term injury-driven glutamatergic changes have not been well characterized, and the sex-specific differences potentially influencing glutamatergic dysregulation post-TBI have been particularly understudied [15,16]. However, recent work has begun to identify chronic changes in glutamatergic neurotransmission and metabotropic glutamate receptor expression after injury, highlighting a critical gap in our understanding of how these changes differ between sexes and contribute to divergent recovery trajectories [16,17,18].

Altered expression of glutamatergic proteins, including N-methyl-D-aspartate (NMDA) receptor subunits and glutamate transporters, has been reported across multiple neuropsychiatric disorders, including major depressive disorder (MDD), bipolar disorder, and schizophrenia [19,20,21,22,23,24,25]. This is particularly relevant given the high prevalence of mental health challenges among patients experiencing prolonged recovery following TBI, highlighting a potential mechanistic link between injury and these disorders. Furthermore, baseline sex differences in both the prevalence and symptomology of mental health disorders may play into divergent, sex-specific TBI outcomes [26,27]. In addition to affective symptoms, memory impairments are common after TBI, and glutamatergic signaling is crucial in memory consolidation and retrieval through NMDA receptor-dependent long-term potentiation, a key mechanism underlying synaptic strengthening and learning [28]. Given the vital role of glutamatergic signaling in cognitive and emotional functions, and the documented sex differences in neuropsychiatric disorders, investigating sex-specific alterations in the glutamate system in association with long-term behavioral deficits following TBI is both timely and highly relevant.

Previous studies using this model have demonstrated a delayed onset of affective-like behavioral deficits, including impaired social interaction and reduced self-care tendencies, associated with changes in glutamatergic protein expression [29]. Building on this work, the current study examined sex differences in glutamate signaling within the prefrontal cortex and hippocampal subregions during the chronic phase following closed-head injury, given the essential role of the corticolimbic system in TBI-related behavioral deficits. In parallel, we aimed to identify sex-specific proteomic signatures related to injury-induced dysregulation of glutamatergic pathways. Proteomic analyses revealed widespread dysregulation of key pathways, cellular processes, and molecular regulators involved in excitatory signaling and synaptic function. Biomarker analysis identified a single shared biomarker between males and females, alongside multiple biomarkers unique to each sex. Consistent with these findings, we observed sex-specific changes in the expression of NMDA receptor subunits and the glutamatergic postsynaptic protein PSD95, suggesting that dynamic and persistent molecular changes occur within the prefrontal cortex and hippocampal subregions of the rat brain three months following a single mild traumatic injury.

## 2. Results

### 2.1. IPA Predicted Dysregulation of Excitatory Signaling Pathways, Behavioral Changes, and Upstream Regulator Activity

**Figure 1:**
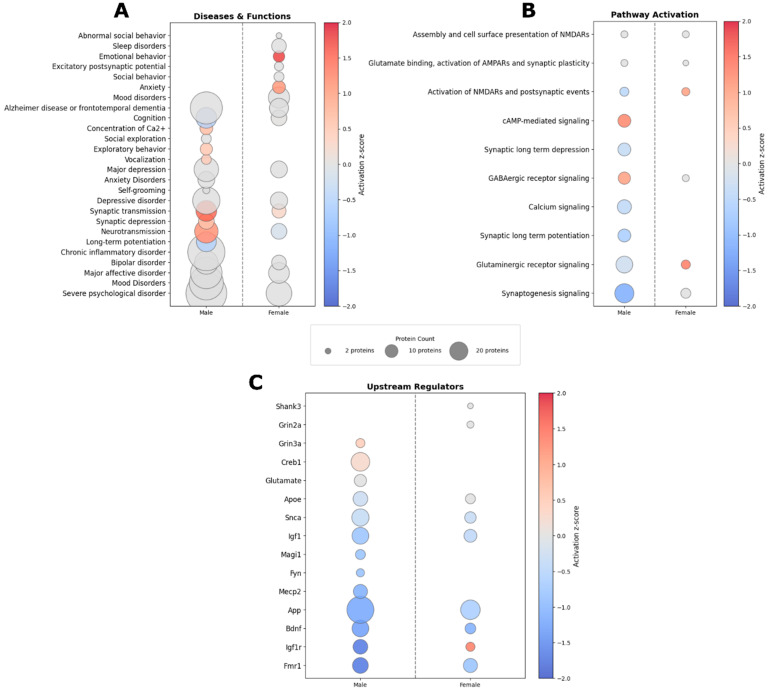
IPA predicted dysregulation of diseases and function, canonical pathways, and upstream regulator molecules in both sexes. Bubble plots illustrate the significantly dysregulated diseases and functions (**A**), pathways (**B**), and predicted upstream regulators (**C**) identified by IPA based on the differentially expressed proteins detected for each sex. The activation state is denoted by the color of the bubble, corresponding to the activation z-score value. Bubble size corresponds to the number of DEPs within each enriched term. Data in the above figure were derived from IPA.

Proteomic analysis revealed significant enrichment of numerous diseases and functional categories based on the differentially expressed proteins (DEPs) in both sexes (Figure 1A). These categories included mood disorders, depression-like behaviors, glutamate signaling-related processes, cognitive performance, and anxiety-like behaviors. Many of the disease and function terms identified by Ingenuity Pathway Analysis (IPA) align with behavioral deficits and symptomology observed in TBI patients, suggesting a potential mechanistic link between the chronic protein expression changes and long-term behavioral outcomes. Notably, several disease and functional categories were dysregulated in a sex-specific manner. Specifically, males showed dysregulation in functions related to social exploration, exploratory behavior, and self-grooming, where these categories were not significantly altered in females. In contrast, females showed dysregulation related to sleep disorders and emotional behaviors, with emotional behavior predicted to be significantly activated following injury.

Pathways related to glutamatergic signaling, synaptic plasticity, and NMDA receptor activity were significantly dysregulated in both sexes following injury (Figure 1B). Although males and females shared enrichment across many glutamatergic and synaptic pathways, several signaling cascades—including synaptic long-term depression, synaptic long-term potentiation, calcium signaling, and cyclic AMP (cAMP)–mediated signaling—were significantly dysregulated exclusively in males. Interestingly, while both sexes exhibited changes to “glutaminergic receptor signaling” and “activation of NMDARs and postsynaptic events,” these pathways were predicted to be activated in females but inhibited in males, highlighting divergent sex-specific regulatory patterns in chronic glutamatergic signaling following injury.

IPA was used to identify upstream regulators of the DEPs in male and female datasets, along with their predicted activation states (Figure 1C). Notably, several common upstream regulator molecules were identified in both males and females, including *Apoe*, *Snca*, *Igf1*, *App*, *Bdnf*, *Igf1r*, and *Fmr1*. These molecules play an essential role in synaptic vesicle cycling, synaptic plasticity, glutamatergic signaling, and postsynaptic organization [30,31,32,33,34,35]. In both sexes, the majority of these upstream regulators were predicted to be inhibited. However, *Igfr1* exhibited a sex-specific pattern, being predicted as inhibited in males but activated in females. These shared upstream regulator activities suggest common elements of chronic TBI pathophysiology that may represent convergent targets for therapeutic intervention.

In contrast, several upstream regulators were uniquely identified in the male dataset, including *Creb1*, *Grin3a*, and glutamate. *Grin3a* (GluN3A) encodes a modulatory NMDA receptor subunit that influences receptor biophysics and synaptic maturation, directly linking it to NMDA receptor structure and function [36,37]. *Creb1* is a canonical activity-dependent transcription factor downstream of NMDA receptor-mediated calcium signaling and regulates expression of synaptic plasticity genes such as *Bdnf*, consistent with a role in injury-induced activity-dependent gene programs [38]. The identification of glutamate itself as an upstream regulator further supports the interpretation that males exhibit changes in proteins downstream of excitatory neurotransmission and NMDA receptor activation during the chronic phase following injury.

Conversely, *Grin2a* and *Shank3* emerged as upstream regulator predictions unique to females. *Grin2a* encodes the GluN2A subunit, a dominant GluN2 subunit component that critically contributes to NMDA receptor function and synaptic localization, and was also evaluated in the immunohistochemical analyses. *Shank3* encodes a major postsynaptic scaffolding protein that organizes PSD95 and NMDA receptors at excitatory synapses, and perturbations in Shank3 signaling alter NMDA receptor surface expression and PSD composition [39]. Together, these upstream regulator predictions support the presence of chronic glutamatergic dysfunction in the frontal cortex of both sexes while highlighting distinct, sex-specific molecular nodes that may drive divergent downstream proteomic signatures following injury.

### 2.2. Sex-Specific DEPs Were Identified Within Commonly Dysregulated Glutamate-Related Signaling Pathways

**Figure 2:**
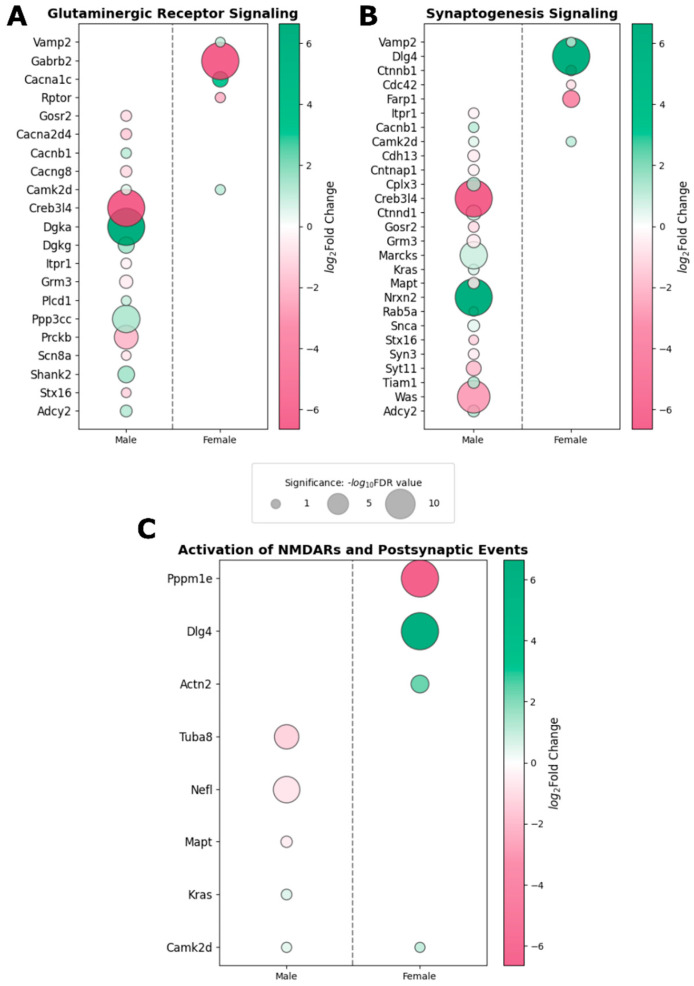
Pathways related to glutamatergic signaling and synaptic remodeling were altered in a sex-specific manner. Bubble plots depict the differentially expressed proteins within the glutaminergic signaling pathway (**A**), the synaptogenesis signaling pathway (**B**), and activation of NMDARs (NMDA receptors) and postsynaptic events pathway (**C**). While these pathways were enriched in both sexes, males and females did not exhibit dysregulation of the same proteins within each pathway. Camk2d expression was altered in all three pathways in both sexes. However, no other overlapping DEPs were present in this subset of enriched pathways. Notably, *Dlg4* (PSD95) was profoundly upregulated in the female frontal cortex. Bubble size represents statistical significance in terms of −log_10_FDR value, and bubble color denotes the directionality and magnitude of the expression change in terms of log_2_fold change. Data in the above figure were derived from IPA.

DEPs associated with glutaminergic receptor signaling (Figure 2A), synaptogenesis signaling (Figure 2B), and the activation of NMDARs and postsynaptic events (Figure 2C) were significantly dysregulated in both sexes. To assess sex-specific patterns within these shared pathways, DEPs were identified in each pathway and compared across sexes to identify common DEPs that changed within these pathways and DEPs unique to each sex. Interestingly, *Camk2d*, which encodes calcium/calmodulin-dependent kinase II, was the only protein consistently altered in both sexes across these enriched pathways, and it was significantly upregulated in both sexes. In contrast, all other DEPs were uniquely changed in either males or females. These findings suggest that although similar glutamatergic and synaptic pathways are disrupted in both sexes following injury, the underlying molecular changes are largely sex-specific. Of particular note, *Dlg4*, which encodes the protein PSD95, was significantly increased in the frontal cortex of females only, further supporting the presence of sex-dependent molecular remodeling of excitatory synapses in the chronic phase following injury.

### 2.3. Biomarker Analysis Uncovered Sex-Specific Translational Biomarkers Within the Frontal Cortex

The biomarker comparison analysis feature within IPA was used to identify potential translational biomarkers within the male and female proteomic datasets. The analysis identified 11 candidate biomarkers in males (Table 1) and 6 in females (Table 2). These candidate TBI biomarkers have been previously validated in the context of at least one disease or clinical condition; however, additional validation of their prognostic significance in TBI will be essential for improving our understanding of injury progression and the heterogeneity of outcome trajectories. Notably, only a single molecule, *Atp6v1e1*, was identified as a shared translational biomarker across both sexes. *Atp6v1e1* encodes a proton-transporting ATPase subunit responsible for acidification of intracellular compartments, a process critical for endocytosis, neurotransmitter loading into synaptic vesicles, receptor recycling, and protein degradation [40,41]. Expression of Atp6v1e1 was increased in both sexes following injury. In contrast, reduced expression of this protein in blood and brain tissue has been reported as a biomarker for Alzheimer’s disease, suggesting that its upregulation after injury may reflect distinct or potentially compensatory mechanisms associated with traumatic neurodegeneration [42,43].

### 2.4. Region-Specific Alterations in NMDA Receptor Subunit Expression Were Accompanied by PSD95 Expressional Changes

**Figure 3:**
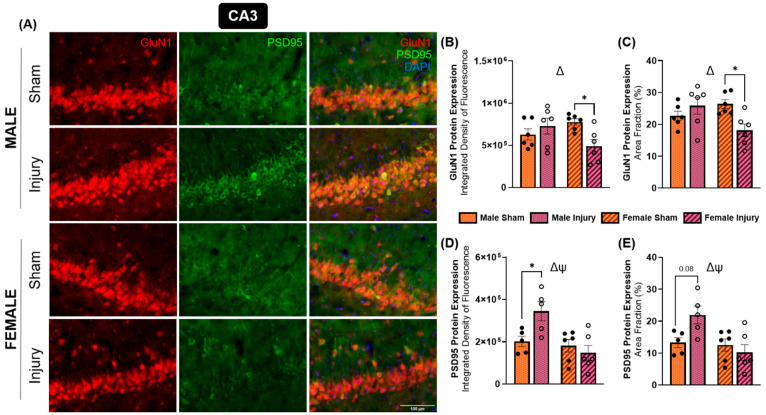
GluN1 and PSD95 expression exhibited sex-dependent alterations in the CA3 hippocampal subregion. Brain tissue was analyzed for immunofluorescent expression of GluN1 and PSD95 in the CA3 in each experimental group (**A**). Integrated density of fluorescence (**B**) and area fraction (**C**) of GluN1 demonstrated significant attenuation in injured female animals. The integrated density of fluorescence (**D**) and area fraction (**E**) of PSD95 were elevated in injured males. * *p* < 0.05, ψ = significant main effect of sex, Δ = significant interaction effect of injury X sex.

For each marker of interest, expression was quantified using two complementary measures: integrated density of fluorescence and the area fraction (percent positively stained area). For each outcome, a two-way ANOVA with factors of injury and sex was conducted to measure changes in protein expression for each marker of interest. Analyses for each marker were conducted in all regions of interest. However, only region–marker combinations that reached statistical significance are presented. All other combinations showed no significant effects and are therefore not shown. A significant interaction between sex and injury was detected in the CA3 hippocampal subregion in terms of both integrated density (*p* = 0.0153) (Figure 3B) and area fraction (*p* = 0.0078) (Figure 3C). The significance of the interaction between these variables demonstrated a sex-specific change in GluN1 protein expression in response to injury. Further, a significant downregulation of GluN1 in injured female animals was detected in both integrated density (*p* = 0.0502) and area fraction (*p* = 0.0317). Because GluN1 is the obligatory subunit of NMDA receptors, these findings imply sex-dependent directionality in chronic NMDA receptor expression within the CA3 subregion, with males exhibiting increased expression and females showing reduced expression following injury.

Similarly, expression of glutamatergic postsynaptic density marker PSD95 displayed sex-dependent alterations in the CA3 subregion. A significant injury and sex interaction effect was observed for both integrated density (*p* = 0.0187) (Figure 3D) and area fraction (*p* = 0.0264) (Figure 3E). Injured males exhibited an increased integrated density of PSD95 fluorescence compared to sham males (*p* = 0.0501), whereas females showed a trend toward reduced PSD95 expression that did not reach significance. Additionally, females showed lower PSD95 expression than males, regardless of injury status, as indicated by both integrated density (*p* = 0.0054) and area fraction (*p* = 0.0127) measurements. Representative immunofluorescence images are shown in Figure 3A.

**Figure 4:**
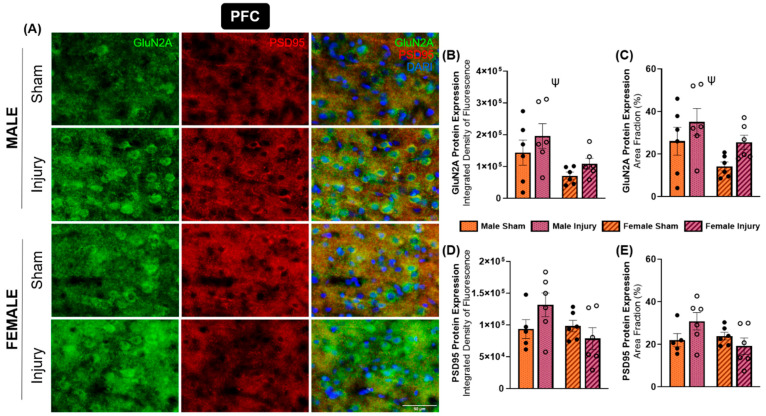
Expression of GluN2A and PSD95 was changed in the prefrontal cortex. Brain tissue was analyzed for immunofluorescent expression of GluN2A and PSD95 in the PFC for each experimental group (**A**). Integrated density of fluorescence (**B**) and area fraction (**C**) of GluN2A showed injury-driven increases in both sexes. The integrated density of fluorescence (**D**) and area fraction (**E**) of PSD95 expression appeared to increase in injured males but decreased in injured females, although not significantly. ψ = significant main effect of sex.

GluN2A is one of the predominantly expressed subunits of the NMDA receptor. This subunit is classically thought to be primarily expressed synaptically, supporting more neuroprotective, pro-survival signaling [44]. In the present study, changes in GluN2A expression were localized to the prefrontal cortex in both sexes (Figure 4A). A significant effect of sex was detected for both integrated density (*p* = 0.0140) (Figure 4B) and area fraction (*p* = 0.0427) (Figure 4C), with females expressing significantly lower GluN2A expression within the prefrontal cortex compared to males. Additionally, the effect of injury on area fraction approached statistical significance (*p* = 0.0521), suggesting a trend toward increased GluN2A expression in the PFC of both sexes following injury.

PSD95 expression was also evaluated in the prefrontal cortex. Analysis revealed an injury-by-sex interaction that approached statistical significance for both integrated density (*p* = 0.0771) (Figure 4D) and area fraction (*p* = 0.0583) (Figure 4E). Although these interactions did not meet the threshold for statistical significance, a clear pattern emerged in which injured males exhibited increased PSD95 expression, whereas injured females showed a modest reduction. Together, these findings suggest sex-dependent differences in synaptic remodeling within the prefrontal cortex following injury.

**Figure 5:**
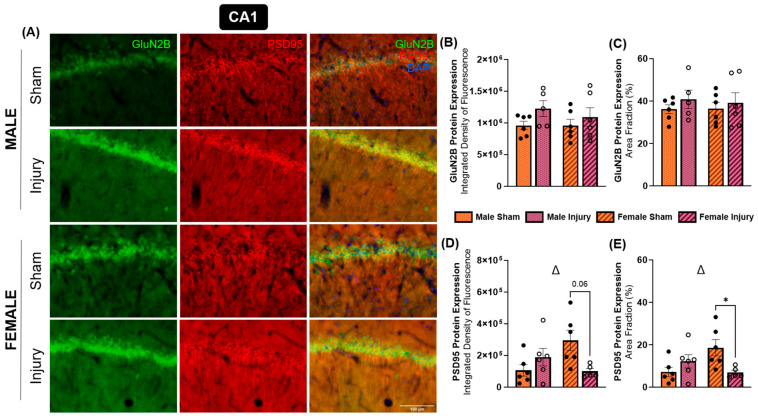
Sex-specific changes in PSD95 expression, but not GluN2B expression, were revealed in the CA1 hippocampal subregion. Brain tissue was analyzed for immunofluorescent expression of GluN2B and PSD95 in the CA1 hippocampal subregion in each experimental group (**A**). Integrated density of fluorescence (**B**) and area fraction (**C**) of GluN2B did not demonstrate any significant expression differences in response to injury. The integrated density of fluorescence (**D**) and area fraction (**E**) of PSD95 expression were significantly downregulated in injured females, and this effect was not seen in males. * *p* < 0.05, Δ = significant interaction effect of injury X sex.

**Figure 6:**
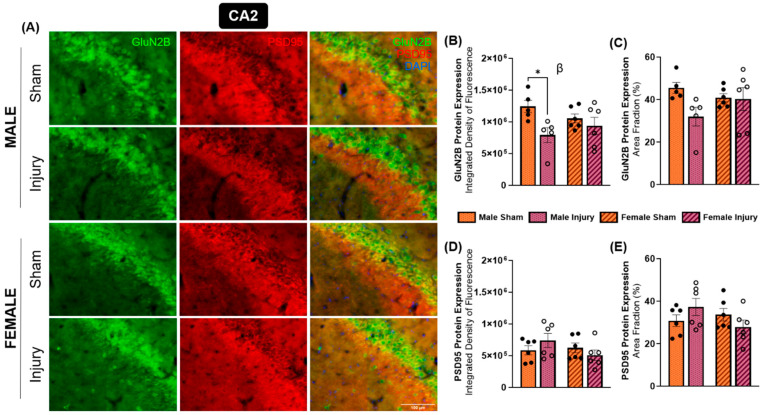
Injury-driven changes were evident in CA2 GluN2B expression, but not PSD95 expression. Brain tissue was analyzed for immunofluorescent expression of GluN2B and PSD95 in the CA2 in each experimental group (**A**). Integrated density of fluorescence (**B**) and area fraction (**C**) of GluN2B exhibited decreases among injured males, but not injured females. PSD95 expression was not significantly altered in terms of integrated density of fluorescence (**D**) or area fraction (**E**). * *p* < 0.05, β = significant main effect of injury.

GluN2B is another highly expressed NMDA receptor subunit in the brain. GluN2B, alongside GluN1 and GluN2A, forms the triheteromeric NMDA receptor, which is thought to constitute a substantial, potentially predominant fraction of NMDA receptors in the central nervous system [45]. In contrast to GluN2A, GluN2B has been reported to be more abundantly expressed at extrasynaptic sites and has been associated with neurodegenerative signaling pathways [44]. At three months post-injury, changes in GluN2B expression were primarily observed in the CA1 and CA2 hippocampal subregions (Figure 5A and Figure 6A). Within the CA1, the effect of injury was approaching significance in terms of integrated density (*p* = 0.0907) (Figure 5B) but not area fraction (Figure 5C). These findings suggest a modest, non-significant increase in CA1 GluN2B expression in both sexes following injury. In contrast, a significant injury effect was detected in the CA2 subregion for integrated density of GluN2B expression (*p* = 0.0171) (Figure 6B), and this effect was approaching significance in area fraction measures (*p* = 0.0850) (Figure 6C). Post hoc Tukey’s multiple comparisons test revealed a significant decrease in GluN2B integrated density in injured males compared to their sham counterparts (*p* = 0.0506) in the CA2 (Figure 6B), an effect that was not observed in females.

PSD95 expression in the CA1 and CA2 subregions also showed sex-specific alterations. Specifically, a significant interaction effect between injury and sex was observed in the CA1 regarding both integrated density (*p* = 0.0104) and area fraction (*p* = 0.0092), indicating sex differences in PSD95 expression changes in the CA1 post-injury. Tukey’s multiple comparisons test showed a decrease in PSD95 expression among injured females compared to shams in integrated density (*p* = 0.0554) (Figure 5D) and area fraction (*p* = 0.0514) (Figure 5E). This effect was faintly observed in the CA2, with an interaction effect approaching significance for area fraction (*p* = 0.0751) (Figure 6E), but no changes were detected in integrated density in the CA2 subregion (Figure 6D).

**Figure 7:**
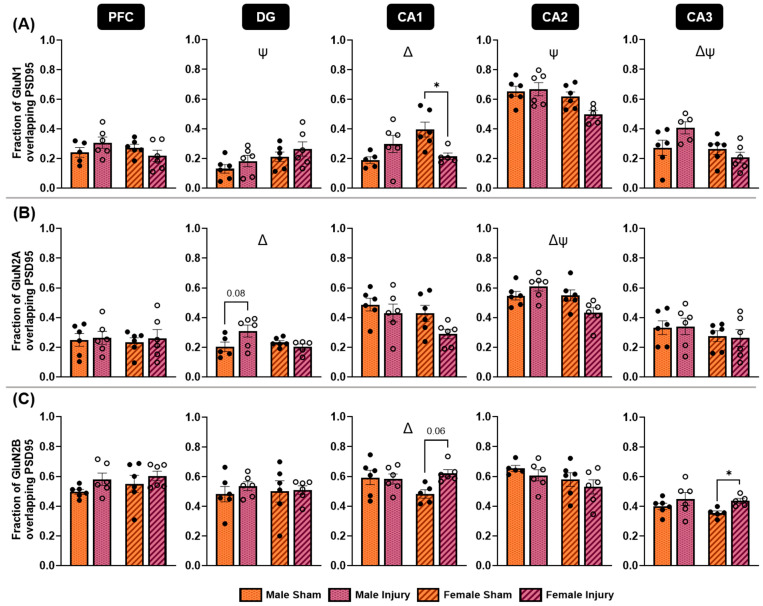
Colocalization of NMDA receptors with PSD95 was altered following injury. Each region of interest was evaluated for changes in the colocalization of each NMDA receptor subunit with PSD95. Localization of GluN1 with PSD95 changed in a sex-dependent manner in all regions of interest, except the DG hippocampal subregion (**A**). This effect was especially evident in the CA1, where females demonstrated a significant decrease in colocalization between the markers following injury, and males did not. Patterns of colocalization for GluN2A (**B**) and GluN2B (**C**) differed across regions, with some effects driven by injury and some by sex differences. * *p* < 0.05, ψ = significant main effect of sex, Δ = significant interaction effect of injury X sex.

Colocalization of each NMDA receptor subunit marker with PSD95 was quantified using the Manders colocalization coefficient. Specifically, the fraction of the NMDA receptor subunit signal colocalized with PSD95 was analyzed within each region of interest. GluN1/PSD95 colocalization was assessed using a two-way ANOVA within each region to compare the effects of injury and sex (Figure 7A). Significant injury-by sex interaction effects were observed in the CA1 (*p* = 0.0047) and CA3 (*p* = 0.0326) hippocampal subregions, with similar trends approaching significance in the PFC (*p* = 0.0900) and CA2 (*p* = 0.0772) regions. Post hoc Tukey’s multiple comparisons revealed a significant decrease in GluN1/PSD95 colocalization in the CA1 among injured females (*p* = 0.0490). Overall, injured males tended to increase colocalization of GluN1 with PSD95 in regions exhibiting significant interaction effects, whereas females tended to decrease colocalization. In contrast, no significant injury-by-sex interaction was detected in the DG; however, this region did show a significant effect of sex (*p* = 0.0449), as did the CA2 (*p* = 0.0094) and the CA3 (*p* = 0.0207). Females showed a higher fraction of GluN1/PSD95 colocalization in the DG compared to males, while the opposite was true for the CA2 and CA3 subregions. Overall, these results suggest that both male and female animals exhibit injury-induced alterations in NMDA receptor localization within overlapping brain regions, but the directionalities of these changes are frequently sex-dependent.

Colocalization of the GluN2A and GluN2B NMDA receptor subunits with PSD95 was assessed to determine whether receptors containing these subunits exhibited altered synaptic localization following injury (Figure 7B,C). No significant differences in colocalization were detected in the PFC for either subunit. In the DG, GluN2A/PSD95 colocalization showed a significant interaction effect (*p* = 0.0298), with injured males increasing colocalization and injured females maintaining levels comparable to controls. In contrast, no significant differences in GluN2B/PSD95 colocalization were detected in the DG. Within the CA1 subregion, GluN2A/PSD95 colocalization showed effects of injury (*p* = 0.0575) and sex (*p* = 0.0502), which approached significance, indicating a potential injury-driven decrease in colocalization in both sexes following injury. In contrast, GluN2B/PSD95 analysis demonstrated a significant interaction between injury and sex (*p* = 0.0499) in the CA1, with injured females showing elevated colocalization, whereas males showed no changes. A significant interaction effect was also detected in the CA2 for GluN2A/PSD95 measures (*p* = 0.0141), in which males increased colocalization, and females decreased it following injury. No significant effect was detected in the CA2 regarding GluN2B/PSD95 colocalization. Lastly, the CA3 showed no significant changes in GluN2A/PSD95 colocalization. However, injured females showed a significant increase in GluN2B/PSD95 colocalization in the CA3 subregion (*p* = 0.0105), as determined by Welch’s *t*-tests corrected for multiple comparisons. Together, these findings reveal region-specific and sex-dependent alterations in NMDA receptor synaptic localization, with distinct patterns emerging between GluN2A- and GluN2B-containing receptor populations. These contrasting localization profiles suggest differential regulation of NMDA receptor subtypes following injury, potentially reflecting divergent mechanisms of synaptic remodeling in males and females.

## 3. Discussion

A substantial proportion of individuals who sustain a TBI go on to develop persistent adverse symptoms that can profoundly disrupt daily functioning. Recovery trajectories following TBI differ between males and females, with females frequently reporting greater symptom severity and a higher likelihood of developing persistent post-injury symptoms. Affective disorders, such as anxiety and depression, are common among patients with persistent symptoms, and some studies suggest that females may be more vulnerable to the development of neuropsychiatric conditions following injury. Despite these clinical observations, the mechanisms underlying these long-term changes are unknown. However, glutamatergic projections interconnect key brain regions involved in affective regulation, including the prefrontal cortex and hippocampus, and alterations to excitatory signaling have been implicated in the pathophysiology of depression.

Consistent with this framework, our immunofluorescence analyses revealed sexually dimorphic responses within the glutamate system following TBI. Expression of PSD95, a core postsynaptic scaffolding protein at glutamatergic synapses, was altered in a sex-specific manner, although not all pairwise comparisons reached statistical significance. The most profound changes were observed in the CA1 and CA3 hippocampal subregions, where females exhibited significantly downregulated expression of PSD95, whereas males demonstrated significantly elevated PSD95 expression in both regions. These findings align with prior work showing that PSD95 expression in males decreases 24 h following injury [46] and remains decreased for several days [47]. In contrast, studies using moderate, but not mild, controlled cortical impact (CCI) models have reported transient increases in PSD95 protein expression during the first week post-injury, followed by normalization by two weeks [48]. Similarly, synaptosomal analyses following CCI showed early increases in PSD95 expression at one day, a decreased expression at one week, and recovery by two weeks, which was maintained through four weeks in both males and females [49]. Together, these findings suggest that PSD95 expression is dynamically regulated following brain injury depending on injury severity, brain region, time post-injury, and biological sex. The chronic, sex-specific alterations observed in the present study point to persistent differences in synaptic remodeling that may contribute to divergent long-term behavioral and affective outcomes following mTBI.

Similar to PSD95, GluN1 expression exhibited sex-dependent alterations following injury, most notably within the CA3 hippocampal subregion. Injured females displayed a significant decrease in expression, whereas males showed no corresponding change. Because GluN1 is the obligatory subunit in all NMDA receptors, this finding suggests that females chronically downregulated NMDA receptors in this region following a single injury. The CA3 region occupies a central position within the hippocampal trisynaptic circuit, receiving input from the dentate gyrus via mossy fibers and projecting to CA1 pyramidal neurons through Schaffer collaterals [50]. Therefore, a downregulation in NMDA receptor expression, and likely NMDA receptor signaling, may suggest functional disruption of this critical circuit.

In contrast to the sex-dependent changes observed for GluN1 in the CA3, differences in GluN2A and GluN2B expression tended to be primarily injury-driven and localized to distinct brain regions. GluN2A expression showed modest increases in injured animals, specifically in the PFC, while GluN2B generally increased in CA1 and decreased in CA2 hippocampal subregions with injury. These injury-induced, region-specific changes in GluN2B expression suggest long-term dysfunction or adaptation of the trisynaptic circuit. Consistent with this interpretation, previous studies have reported sustained alterations in postsynaptic signaling within the hippocampus up to four weeks following CCI, including sex- and injury-dependent changes in the expression and phosphorylation of proteins involved in long-term potentiation [49]. Furthermore, prolonged desensitization of NMDA receptors has been proposed to occur following the acute period of overactivation following injury [51], which may be related to modifications in NMDA receptor subunit expression. However, the duration and functional consequences of this desensitization remain incompletely understood.

Patterns of colocalization between NMDA receptor subunits and PSD95 were chronically altered following TBI. For GluN,1 colocalization with PSD95 displayed pronounced sex-specific alterations, with injured males showing increased colocalization and females exhibiting decreased colocalization. Importantly, these trends were observed across regions, with the exception of the DG, suggesting a more widespread change rather than a region-specific one. As regions of interest included both the neuronal cell bodies and dendrite-rich areas, these changes in colocalization likely reflect a combination of altered synaptic localization and shifts in intracellular distribution. Further, the observed patterns may be influenced by regional changes in PSD95 expression, particularly given that GluN1 expression was altered only in the CA3.

PSD95 is known to form a signaling complex with NMDA receptors, particularly those containing the GluN2B subunit, and neuronal nitric oxide synthase (nNOS). This molecular assembly plays a critical role in excitotoxic cell death [52]. Inhibiting the interaction between NMDA receptors and PSD95 following TBI has been found to promote functional recovery [46], highlighting the functional relevance of receptor–scaffold coupling. Accordingly, the sex-specific differences in NMDA receptor–PSD95 colocalization observed in the present study may have significant consequences for downstream signaling pathways and long-term cellular outcomes. To our knowledge, this study represents the first systematic evaluation of sex-dependent changes in colocalization of PSD95 with NMDA receptor subunits at chronic time points following injury. Future studies targeting synaptosomal fractions within the prefrontal cortex and hippocampal subregions will be essential to more precisely define synaptic NMDA receptor expression and function after TBI and to determine how biological sex modulates these processes.

Despite males exhibiting significant chronic dysregulation in more than twice as many proteins as females, IPA revealed striking convergence in the disease categories, biological functions, and signaling pathways affected by injury in both sexes. Pathways associated with neurotransmission and intracellular signaling cascades were significantly enriched in both datasets, with glutamatergic signaling, synaptogenesis signaling, and activation of NMDARs and postsynaptic events emerging as shared key pathways. Glutamatergic dysfunction has been implicated in the pathophysiology of post-mTBI depression [53], a condition frequently reported among individuals with persistent concussion symptoms [54,55]. NMDA receptors play a critical role in long-term potentiation signaling through the initiation of calcium-dependent secondary messenger cascades, all of which are central to proper learning and memory processes [28,37]. In alignment with our findings, Morin et al. reported chronic (24 weeks) activation of signaling pathways related to long-term potentiation, calcium activity and synaptogenesis in the cortex following repeated closed-skull mTBI in mice, although sex-specific effects were not examined [56].

In the present study, while many of the same biological pathways were enriched in both sexes, there was minimal overlap in the specific proteins contributing to these effects. Within the glutamatergic synapse, synaptogenesis signaling, and activation of NMDARs and postsynaptic events pathways, only a single protein was commonly dysregulated across sexes. This suggests that although comparable neurobiological processes are affected by injury in males and females, they are likely mediated through distinct molecular mechanisms. This divergence highlights the importance of incorporating sex-specific considerations into therapeutic strategies aimed at mitigating injury-induced dysfunction. Consistent with these findings, sex-dependent regulatory networks associated with the DEPs were identified. Key mediators of glutamatergic signaling were predicted to be altered in both sexes, with *Creb1*, *Grin3a*, and glutamate identified in males, and *Grin2a* and *Shank3* in females. Additionally, multiple upstream regulators associated with synaptic signaling, postsynaptic structure, and plasticity were predicted to be dysregulated in both datasets. These observations are consistent with the pathway enrichment analyses, which indicate dysregulation of glutamatergic signaling and NMDA receptor activity, and further support the sex-specific expression patterns observed in our immunofluorescent analyses.

Biomarker comparison analysis using IPA further identified candidate translational biomarkers within the male and female proteomic datasets. This approach revealed 11 potential biomarkers in males and 6 in females, with only one molecule, Atp6v1e1, shared between sexes. Atp6v1e1 is a proton-transporting ATPase subunit that mediates acidification of intracellular compartments, a process essential for endocytosis, neurotransmitter loading into synaptic vesicles, receptor recycling, and protein degradation [40,41]. Expression of Atp6v1e1 was elevated in both sexes following injury, suggesting a sustained adaptive or compensatory response to altered vesicular or lysosomal function. Notably, decreased expression of Atp6v1e1 in both blood and brain tissue has been reported as a potential biomarker for Alzheimer’s disease [42,43], further supporting its relevance to neurodegenerative and injury-related mechanisms. Strikingly, aside from Atp6v1e1, all candidate biomarkers were sex-specific. In males, the biomarker profile suggested processes related to axonal degeneration, metabolic dysfunction, and altered cellular signaling mechanisms. Specifically, downregulation of both Mbp and Mapt pointed to chronic demyelination and cytoskeletal instability following injury, as these proteins are critical to axonal integrity [57,58]. Disruption of cellular energy metabolism was suggested by the downregulation of Ckm, a kinase involved in energy homeostasis and ATP buffering, and upregulation of lysosomal enzyme Gaa [59,60]. Furthermore, upregulation of Snca and Chga suggests persistent signaling alterations in synaptic vesicle trafficking and neuroendocrine signaling, respectively [61,62]. In stark contrast, the candidate biomarkers identified in females suggested mechanisms more closely associated with glial activation and structural remodeling. Elevated expression of Gfap at this chronic time point strongly suggests persistent astrocyte reactivity and sustained neuroinflammation [63]. Concurrent upregulation of Actn2 and Dtna indicates potential adaptations in cytoskeletal organization and synaptic stabilization in females [64,65], contrasting with the structural instability implied by the male proteomic profile. Together, these findings highlight striking sex-specific divergences not only in the candidate biomarkers but also in the biological mechanisms potentially underlying their expressional changes. These differences underscore the importance of further investigations into sex-dependent molecular responses to TBI. At the same time, the identification of Atp6v1e1 as a shared translational biomarker suggests that convergent molecular signatures may exist across sexes despite divergent injury-induced proteomic landscapes, supporting its potential utility in developing blood-based diagnostic or prognostic indicators for chronic post-TBI pathology.

This study is subject to several limitations, including the use of separate animal cohorts for immunohistochemical and proteomic analyses, which may introduce variability between outcome measures. Proteomic analyses were confined to the frontal cortex, and future studies extending proteome-level assessments to the hippocampus at a chronic time point would be valuable. Additionally, the inclusion of functional measures of glutamatergic signaling, such as in vivo assessments of glutamate efflux or electrophysiological metrics, would allow for more comprehensive characterization of the chronic glutamatergic alterations observed in the current study. Nonetheless, the results provide compelling preliminary evidence of chronic, sex-specific, and regionally distinct alterations within the glutamate system following TBI. Importantly, these molecular and cellular modifications were observed in brain regions that govern higher-order cognitive and affective functions, suggesting that glutamatergic dysfunction may be a potential mechanistic driver of sex-dependent variability in long-term behavioral outcomes after injury.

Collectively, these findings underscore the need for more integrated and longitudinal investigations into the temporal and functional dynamics of glutamatergic synapses following TBI, with careful consideration of biological sex. Developing a deeper mechanistic understanding of these processes will be critical for identifying sex-informed therapeutic targets and advancing precision-based interventions aimed at mitigating the chronic cognitive and neuropsychiatric sequelae of TBI.

## 4. Materials and Methods

### 4.1. Animals

All procedures were conducted following approval from the Virginia Tech Institutional Animal Care and Use Committee. A random number generator was used to randomly assign animals to the sham or TBI groups, and potential confounders were minimized by ensuring all cages were evenly distributed across shelves to reduce location-related variability. A priori exclusion criteria consisted of skull fracture, mortality, or loss of >20% of pre-injury body weight, as these constituted the humane endpoints of the study. Animals were monitored weekly following injury for signs of pain or distress. No animals were excluded from the study. All procedures were conducted in a vivarium procedure room.

Experiment 1 (Proteomic Analysis): Proteomic data were derived from two cohorts of animals. Cohort 1 consisted of adult, 12-week-old wildtype male Sprague-Dawley (SD) rats (Envigo; Dublin, VA, USA; RRID:RGD_737903) that were randomly divided into two groups: sham (*n* = 3) and injury (*n* = 3). Cohort 2 consisted of 12-week-old wildtype female SD rats, randomly divided into two groups: sham (*n* = 4) and injury (*n* = 4). Rats were acclimated for two weeks on a 12-12 h light dark cycle and were maintained at 70 °F (21 °C) and 70% humidity with ad libitum access to food and water. Wooden blocks and sticks were placed in each cage for enrichment.

Experiment 2 (Immunohistochemistry): Adult, 12-week-old, wildtype male and female SD rats (*n* = 24 animals total) were randomly divided into two groups: sham (*n* = 6 per sex) and injury (*n* = 6 per sex). Acclimation, lighting, environmental conditions, and food/water access were the same as Experiment 1.

### 4.2. Closed-Head Controlled Impact

**Figure 8:**
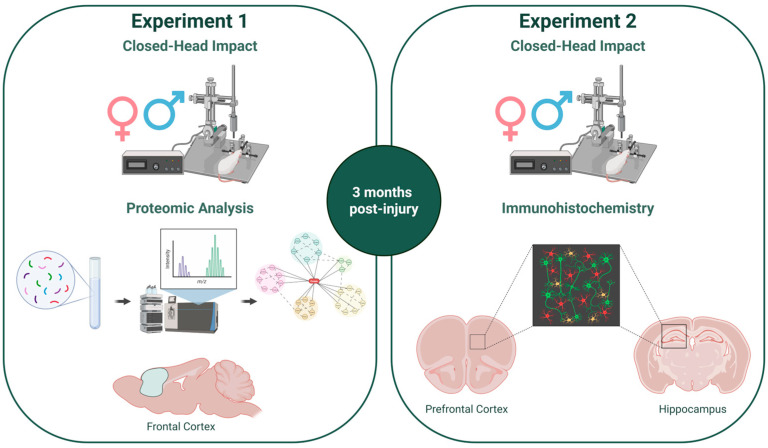
Experimental design. A single closed-head controlled impact was administered to adult male and female rats under isoflurane anesthesia. Three months post-injury, fresh frontal cortex tissue was collected in Experiment 1 for proteomic analysis and fixed brain tissue was collected in Experiment 2 for immunohistochemical analysis. Created in BioRender. VandeVord, P. (2026) https://BioRender.com/el4otaa (accessed on 3 February 2026).

A closed-head controlled impact was used as a clinically relevant model of mTBI [66]. The protocol was carried out as described previously [67,68]. Briefly, animals were anesthetized under 4% isoflurane for 5 min before being placed atop a 1.25 cm thick foam pad in the stereotaxic frame (Harvard Apparatus, Holliston, MA, USA; cat# 75-1806). The front teeth were affixed to the bite bar, and a nose cone was secured to continue isoflurane sedation. The skull was not secured with ear bars to allow head movement upon impact and prevent ear injury. The head was shaved, and a rat skull template was used to mark the injury site over the right parietal lobe. An electromagnetic actuator (Neuropactor, Neuroscience Tools, O’Fallon, MO, USA; RRID:SCR_024868) with a flat metal impactor tip 5 mm in diameter was mounted on the stereotaxic crossbar and connected to a control box (Impact One, Leica Biosystems, Buffalo Grove, IL, USA; RRID:SCR_025114). The impactor tip was then firmly zeroed against the skin at the marked injury site. The impactor delivered an injury at a velocity of 6 m/s, a depth of 3 mm, and a dwell time of 300 ms. Immediately following impact, animals were removed from anesthesia and placed in the supine position on a heating pad at 37 °C until they righted themselves. Sham animals were exposed to identical procedures but were not subjected to the impact. This impact method was used for animals in Experiment 2 and for the female cohort in Experiment 1. The male cohort of Experiment 1 was injured as previously described, using a Leica Impact One actuator [29]. The experimental design is displayed in Figure 8.

### 4.3. Tissue Collection and Sample Preparation

Experiment 1: Animals were euthanized by decapitation under 4% isoflurane at three months post-injury. The brain was quickly extracted and rinsed with ice-cold 0.9% saline solution. Following this, the right frontal cortex was dissected and immediately placed in a cooler containing dry ice until samples could be transferred to a freezer at −80 °C for long-term storage.

Protein lysate was isolated from frontal cortex tissue as previously described [29,69]. Protein lysate was stored at −80 °C until preparation for LC-MS/MS procedures. Trypsin digestion was used to cleave proteins into peptides for proteomic analysis. Details of this trypsin digestion method have been published previously [70]. Peptide concentration was determined by measuring the absorbance at 215 nm using a DS-11 FX+ spectrophotometer/fluorometer (DeNovix, Wilmington, DE, USA). Samples were diluted to a concentration of 0.5 mg/mL using solvent A (2:98 LC/MS grade acetonitrile: LC/MS grade water supplemented with 0.1% (*v*/*v*) formic acid). 8 µL (4 µg) of each sample were analyzed using LC-MS/MS (Orbitrap Fusion Lumos, Thermo Fisher Scientific, Waltham, MA, USA), with each sample analyzed twice to yield technical duplicates. Optima™ LC/MS grade solvents and Pierce™ trypsin protease (MS grade) were obtained from Thermo Fisher Scientific (Waltham, MA, USA). LC-MS/MS analysis was conducted by a researcher blinded to the experimental grouping of the animals.

Experiment 2: At three months post-injury, animals were transcardially perfused with ice-cold 0.9% saline followed by 4% paraformaldehyde (PFA). Brains were extracted and stored in 4% PFA at 4 °C overnight, then transferred to 30% sucrose in 1X PBS for 3–4 days at 4 °C. Brains were then embedded in Optimal Cutting Temperature (OCT) compound and stored at −80 °C until cryosectioning. Brains were sliced coronally at a thickness of 30 µm.

### 4.4. Proteomic Analysis

DEPs were identified with an FDR ≤ 0.05. By applying the additional cutoff criteria of fold change (FC) ≥ |1.2| and an adjusted *p*-value ≤ 0.1, we identified 481 DEPs in males and 154 DEPs in females. These thresholds were selected in accordance with previous proteomic studies to capture proteins exhibiting biologically relevant changes in expression while maintaining statistical rigor [70,71]. IPA was then used to examine predicted dysregulation across canonical pathways, regulatory mechanisms, and associated diseases and functions based on the identified DEPs. For each significantly enriched term (FDR value < 0.05), an activation z-score was calculated, and z ≥ |2| was considered significantly activated or inhibited, consistent with the standard IPA threshold indicating strong concordance between observed protein expression patterns and predicted pathway activation states. Additional details regarding the statistical framework underlying IPA analyses have been described previously [72]. Biomarker analysis was performed in IPA to identify translationally relevant biomarkers among the DEPs in each sex. The following characteristics were used as filters to identify translational biomarkers: proteins detected in the blood plasma or serum, cerebrospinal fluid (CSF), saliva, sputum, synovium, tears or urine of humans, rats, or mice, and that have been used as biomarkers for a disease or clinical condition.

### 4.5. Immunofluorescent Staining

Immunofluorescence was used to identify glutamatergic proteins: NMDA receptor subunits GluN1, GluN2A, and GluN2B, and glutamatergic postsynaptic marker PSD95. Each NMDA receptor subunit marker was co-stained with PSD95 to assess changes in the colocalization of these markers. Sections were washed three times for five minutes each in 1X PBS before being permeabilized in 1X PBS + 0.3% Triton-X for 30 min. Sections were then blocked in 2% bovine serum albumin in 1X PBS + 0.3% Triton-X for one hour before overnight incubation with the primary antibody at 4 °C. Primary antibodies were diluted in the blocking buffer solution as indicated in Table 3. After incubation with primary antibody solution, sections were washed three times for five minutes each in 1X PBS + 0.3% Triton-X. Sections were incubated for 1.5 h in secondary antibody solution, with antibodies diluted in blocking buffer as listed in Table 3. Lastly, sections were washed three times for five minutes each in 1X PBS. Samples were stained with DAPI (1:10,000 in 1X PBS) to visualize cellular nuclei, then mounted on microscope slides and coverslipped.

### 4.6. Image Acquisition and Analysis

Brain sections containing the prefrontal cortex and the dorsal hippocampus (DG, CA1, CA2, and CA3) were imaged using an Olympus VS200 Slide Scanner (Olympus Life Sciences, Tokyo, Japan; RRID:SCR_024783) at a magnification of 40×. Virtual slide images were analyzed using QuPath software version 0.5.1 (RRID:SCR_018257), where regions of interest were defined for analysis and exported to ImageJ (Version 1.53k, NIH, Bethesda, MD, USA; RRID:SCR_003070). The integrated density of fluorescence and area fraction were measured for each marker in ImageJ. Additionally, the Just Another Colocalization Plugin (JACoP) (RRID:SCR_025164) for ImageJ was used to analyze colocalization between NMDA receptor subunits and PSD95, specifically to calculate the Manders Overlap Coefficient (MOC). Duplicate sections were analyzed for each animal in each region of interest, and each hemisphere was imaged separately. The values from these four images were averaged for each animal prior to statistical analysis. Immunofluorescence analysis was conducted by a researcher blinded to the experimental grouping of the animals.

### 4.7. Immunofluorescence Statistical Analysis

All immunofluorescence statistical analyses were performed using GraphPad Prism 10 (GraphPad Software, RRID:SCR_002798). Each dataset was tested for outliers by calculating studentized residuals, and data points with studentized residuals outside the range of −2 to 2 were considered outliers. Normality and equal variance assumptions were then tested using the Shapiro–Wilk and F tests, respectively. Datasets that met these assumptions were analyzed using an unpaired *t*-test or a two-way ANOVA to measure differences between sham and injury groups. Datasets that failed to meet normality or equal variance assumptions were either transformed to meet these assumptions or analyzed using a Mann–Whitney U test, Welch’s *t*-test, or multiple unpaired *t*-tests (corrected for multiple comparisons). Post hoc tests were run in the event of a significant ANOVA result.

## Figures and Tables

**Table 1:** Translational biomarkers identified in the male dataset.

Gene Symbol	Family	Log_2_FC	Adj. *p* Value	Protein Function *
*Apoa1*	transporter	6.644	3.30 × 10^−16^	Comprises the primary component of high density lipoprotein (HDL); promotes efflux of cholesterol from tissues
*Atp6v1e1*	transporter	0.456	4.28 × 10^−3^	Acidifies intracellular compartments to influence receptor-mediated endocytosis, protein sorting, and synaptic vesicle processes
*Chga*	other	6.644	3.30 × 10^−16^	Precursor to biologically active peptides that act as neuroendocrine system modulators
*Ckm*	kinase	−0.492	5.72 × 10^−4^	Involved in energy homeostasis through catalysis of phosphate transfer between ATP and phosphogens
*Cyth1*	other	6.644	3.30 × 10^−16^	Plays a role in protein sorting and membrane trafficking
*Gaa*	enzyme	6.644	3.30 × 10^−16^	Responsible for the degradation of glycogen to glucose in lysosomes
*Hba2*	enzyme	0.629	7.72 × 10^−2^	Transports oxygen from the lungs to peripheral tissues
*Mapt*	other	−0.492	3.41 × 10^−2^	Promotes the assembly and stability of microtubules; involved in neuronal polarity
*Mbp*	other	−0.737	2.20 × 10^−3^	Significant component of the myelin sheath in oligodendrocytes and Schwann cells
*Serpina1*	other	0.584	5.56 × 10^−5^	Serine protease inhibitor
*Snca*	enzyme	0.387	2.60 × 10^−2^	Plays a role in membrane trafficking and integration of presynaptic signaling

* Protein functions were derived from GeneCards. ATP = adenosine triphosphate

**Table 2:** Translational biomarkers identified in the female dataset.

Gene Symbol	Family	Log_2_FC	Adj. *p* Value	Protein Function *
*Actn2*	transcription regulator	2.327	2.49 × 10^−4^	Involved in the binding of actin to the cell membrane along microfilament bundles and adherens-type junctions
*Atp6v1e1*	transporter	1.28	9.02 × 10^−4^	Acidifies intracellular compartments to influence receptor-mediated endocytosis, protein sorting, and synaptic vesicle processes
*Comt*	enzyme	−6.644	2.38 × 10^−16^	Metabolizes catecholamines by catalyzing the transfer of a methyl group from S-adenosylmethionine to neurotransmitters including dopamine, epinephrine, and norepinephrine
*Ctsd*	peptidase	−1.718	1.40 × 10^−6^	Responsible for intracellular protein breakdown; involved in APP processing and thought to be involved in the pathogenesis of breast cancer and Alzheimer’s disease
*Dtna*	other	6.644	2.38 × 10^−16^	Component of the dystrophin-associated protein complex; may influence synaptic formation and stability of synapses
*Gfap*	other	0.809	8.80 × 10^−2^	Acts as the major intermediate filament protein in mature astrocytes; elevated expression is associated with astrocytic reactivity

* Protein functions were derived from GeneCards. APP = amyloid-beta precursor protein

**Table 3:** Primary and secondary antibodies used in immunofluorescent staining procedures.

Antibody Name	Product Information	Dilution
Rabbit anti-GluN1	Millipore Sigma (Burlington, MA, USA); cat#: G8913; RRID:AB_259978	1:100
Rabbit anti-GluN2A	Millipore Sigma; cat#: M264; RRID:AB_260485	1:100
Mouse anti-PSD95	Thermo Fisher Scientific; cat# MA1045; RRID:AB_325399	1:250
Mouse anti-GluN2B	BD Biosciences (Franklin Lakes, NJ, USA); cat# 610416; RRID:AB_397796	1:100
Rabbit anti-PSD95	Cell Signaling Technology (Danvers, MA, USA); cat# 3450; RRID:AB_2292883	1:250
Alexa Fluor 546 goat anti-rabbit	Thermo Fisher Scientific; cat# A-11010; RRID:AB_2534077	1:500
Alexa Fluor 488 donkey anti-mouse	Thermo Fisher Scientific; cat# A-21202; RRID:AB_141607	1:500 (1:375 for GluN2B)

## Data Availability

Immunofluorescence data: The original data presented in the study are openly available in Open Data Commons for Traumatic Brain Injury (ODC-TBI; RRID:SCR_021736) at https://doi.org/10.34945/F5Z31P in the dataset entitled “Evaluating Chronic Region- and Sex-Specific Changes in Glutamatergic Proteins in Male and Female Sprague Dawley Rats Following Closed-Head Impact Traumatic Brain Injury Over the Right Parietal Cortex” [73]. Proteomics data: The raw data supporting the conclusions of this article will be made available by the authors on request.

## Abbreviations

The following abbreviations are used in this manuscript:

TBI	Traumatic brain injury
mTBI	Mild traumatic brain injury
DEPs	Differentially expressed proteins
FC	Fold change
IPA	Ingenuity Pathway Analysis
SD	Sprague-Dawley
NMDA	N-methyl-D-aspartate
MDD	Major depressive disorder
cAMP	Cyclic adenosine monophosphate
PSD	Postsynaptic density
DG	Dentate gyrus
CA1	Cornu ammonis 1
CA2	Cornu ammonis 2
CA3	Cornu ammonis 3
CCI	Controlled cortical impact
nNOS	Nitric oxide synthase
FDR	False discovery rate
PBS	Phosphate-buffered saline
OCT	Optimal Cutting Temperature
LC-MS/MS	Liquid chromatography tandem mass spectrometry
PFA	Paraformaldehyde
CSF	Cerebrospinal fluid
